# Posterior fossa extradural haematoma with cerebral venous sinus thrombosis precipitates haemophilia a diagnosis: a paediatric case report and literature review

**DOI:** 10.1007/s00381-025-07040-8

**Published:** 2025-12-02

**Authors:** Melika Akhbari, William Owen, Susan Isabel Honeyman, Saket Badle, Amedeo Calisto

**Affiliations:** 1 Department of Paediatric Neurosurgery, The Children’s Hospital, John Radcliffe Hospital, Oxford University Hospitals NHS Foundation Trust, Oxford, UK; 2 Haemostasis Unit, The Children’s Hospital, John Radcliffe Hospital, Oxford University Hospitals NHS Foundation Trust, Oxford, UK; 3 Nuffield Department of Clinical Neurosciences, University of Oxford, Oxford, UK

**Keywords:** Extradural, Posterior fossa, Haematoma, Haemophilia, Cerebral venous sinus thrombosis, Paediatric neurosurgery, Coagulopathy, TBI

## Abstract

**Background:**

Posterior fossa (PF) fractures are often associated with cerebral venous sinus thrombosis (CVST) yet rarely require treatment beyond anticoagulation. When observed with synchronous, atypical extradural haematoma (EDH) over the transverse sinuses, the management is more equivocal. We present a rare paediatric case of traumatic PF EDH with CVST incidentally unveiling an inherited bleeding disorder (IBD).

**Observations:**

A 3-year-old boy presented following an unwitnessed head injury. Serial imaging confirmed marginal growth of the EDH with stable CVST and an undisplaced occipital skull fracture. A clotting profile sent via PICU admission bloods informed a new diagnosis of haemophilia A. Conservative management monitored EDH evolution against operative risks over the sinus with a clotting disorder. Haematological treatment navigated the quandary of FVIII replacement to prevent bleeding against the need for CVST anticoagulation. The latter was not initiated. A full recovery was made without neurological deficit.

**Conclusions:**

A unifying diagnosis for these incongruent radiological features presented distinct diagnostic and therapeutic challenges. Haematological screening can be misleading in paediatric patients with unclear implications as a diagnostic measure. Excluding coagulopathies is more significant if neurosurgical intervention is indicated, evaluated against individual clinical correlates and symptomatology. In the absence of standardised guidelines for the management of traumatic paediatric EDH with intercurrent CVST and IBDs, the case presented an invitation to thought.

## Introduction

Haemophilia A is a clinical syndrome characterised by an inherited deficiency of factor VIII (FVIII). The severity of symptoms ranges from mild to severe and usually correlates with the amount of FVIII present. There is an increased tendency for bleeding often discordant to the severity of trauma sustained. Intracranial haemorrhage (ICH) is amongst the most serious sequelae, associated with significant morbidity and mortality in a bimodal distribution affecting children < 2 years and adults > 60 years [1]. Extradural bleeding is atypical with the most common site involving the intraparenchymal, subdural or intraventricular space, the latter predominantly in neonates [2, 3].

Paediatric cerebral venous sinus thrombosis (CVST) is rare with an incidence of less than 1 case per 100,000 children annually [4]. Fewer than 5% of cases were implicated following head injury [5] with other causes including inflammatory or infectious aetiologies, malignancy or an idiopathic nature [6]. Indicative symptoms are typically non-specific including seizure, headache and vomiting, leading to delayed or misdiagnosis [7, 8].

There is a paucity of research exploring a relationship between intracranial haemorrhage, CVST and inherited bleeding disorders (IBDs).

We present a paediatric case of acute posterior fossa extradural haematoma (EDH) associated with cerebral venous sinus thrombosis (CVST) facilitating a new diagnosis of haemophilia A. The literature on the topic is summarised.

The rarity of the presentation stemmed from intercurrent intracranial findings raising competing priorities. The incidence of EDH in paediatric head injury is low (2–3%) and even more infrequent in the posterior fossa (1.3% of all head injuries) [9]. Posterior fossa (PF) EDH is considered more sinister, associated with late but rapidly fatal decompensation once a critical threshold for comparatively small volume haematoma is surpassed, often accompanied by delayed neurological deficit. Expansion in the tight PF can lead to brainstem compression, tonsillar herniation and obstructive hydrocephalus [10].

Defining the risk of bleeding was not straightforward. The need for EDH stability balanced against the timely initiation of anticoagulation for CVST and factor replacement for an IBD resulted in conflicting management plans.

This review aims to increase awareness of this rare presentation with emphasis placed on the diagnostic and therapeutic challenges posed by balancing bleeding risk against mitigating further clot propagation.

## Exemplary case description

A 3-year-old, right-handed boy sustained a non-witnessed head injury after falling backwards whilst riding his tricycle without a helmet. Mild subgaleal swelling was noted by his parents following an immediate interval of crying. There was no associated loss of consciousness nor seizure activity. No visual disturbance or focal neurological deficit was observed.

The child was previously fit and well, born at 41 weeks via natural delivery. Parents confirmed that all due vaccinations had been received. No history of abnormal bleeding or bruising had been encountered. All developmental milestones were duly met.

**Fig. 1:**
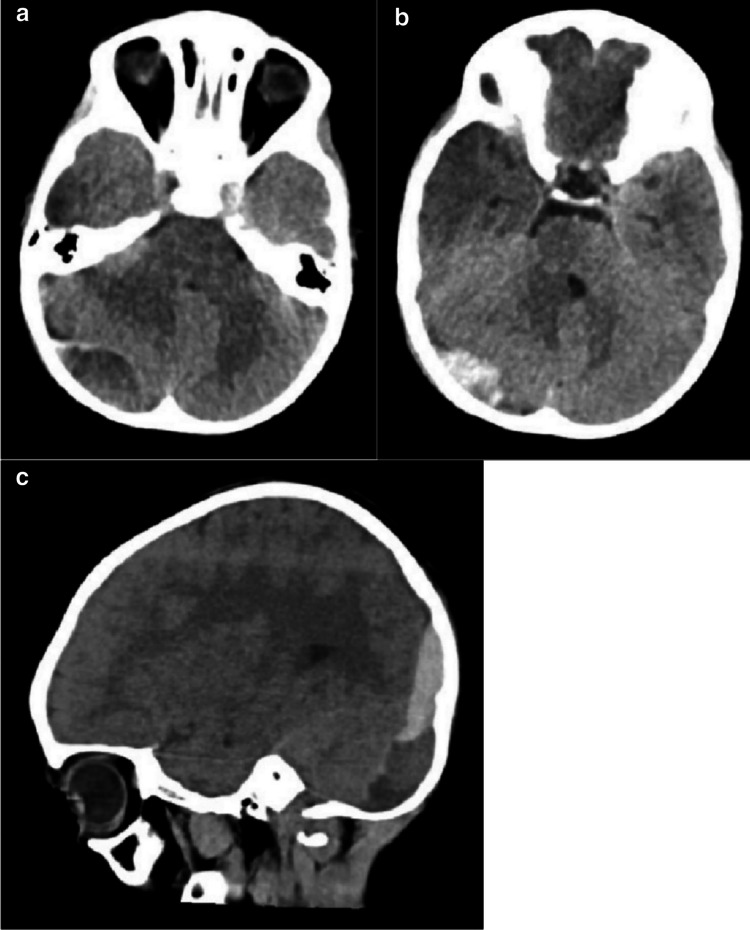
**a** and **b** Axial and **c** sagittal non-contrast enhanced CT images of acute extradural haematoma in the right posterior fossa on presentation.

The patient was noted to be drowsier the next morning with nausea, four episodes of vomiting, lethargy and reduced oral intake. The GCS remained 14–15 (E3V5M6). A CT scan of the head demonstrated a large EDH in the right PF (Fig. 1). This spanned the supra- and infra-tentorial compartments with an acute component in the former and small foci of haemorrhage adjacent to the right cerebellar hemisphere. Transfer to our neurosurgical unit was arranged for further assessment and observation over the course of a 20-day total inpatient stay.

## Management

**Fig. 2:**
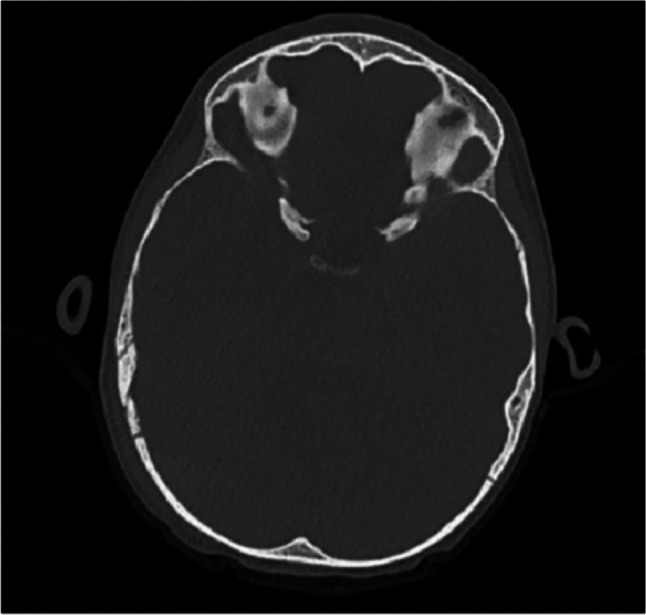
CT bone window of right occipital bone fracture extending to lambdoid and occipitomastoid sutures. The fracture underlies the right subgaleal haematoma, is superiorly undisplaced and inferiorly mildly disrupts the minimally widened lambdoid suture on the right.

Repeat CT Head (CTH) and CT venogram (CTV) at a 5-h interval confirmed stable intracranial appearances. An associated mass effect with effacement of the right occipital lobe and right cerebellar hemisphere and early effacement of the fourth ventricle was noted without supratentorial hydrocephalus at this point. The volume of haemorrhage was predominantly supratentorial. Hyperdensity within the right sigmoid sinus with reduced opacification was concerning for adjacent dural venous sinus thrombosis. Marked expansion of the right sigmoid sinus was noted, although opacification was maintained at the level of the jugular bulb. Compression of the right transverse sinus by the EDH was observed. A right-sided undisplaced occipital skull fracture underlying the right subgaleal haematoma was also noted (Fig. 2).

Given the high risk of neurological decompensation, admission to the paediatric intensive care unit (PICU) was arranged. An incidental finding of elevated APTT sent via admission blood tests was identified, prompting haematology input and extended investigations including factor assays, in view of the atypical EDH appearance. This informed a new diagnosis of mild haemophilia A (baseline FVIII 0.06–0.12 IU/mL or 6–12% consistency).

Subsequent management of the head injury with input from the PICU and haematology teams was deemed nuanced. The risk of an expanding haematoma competing with the need for anticoagulation to mitigate increased thrombotic burden was deliberated. A decision was made not to proceed with immediate MRI under GA to maintain access to the neuroprognostic value of consciousness in the first 72 h following injury. Repeat CTH and CTV at 48 h confirmed marginal growth of the lower attenuation infratentorial fluid component adjacent to the right cerebellar hemisphere (~ 3 mm increase in depth) with an increase in associated PF mass effect and early supratentorial hydrocephalus. A markedly expanded sigmoid sinus persisted on CTV with no thrombus progression. Consensus MDT discussions favoured bleed treatment in the form of factor replacement taking precedence over thrombosis.

**Fig. 3:**
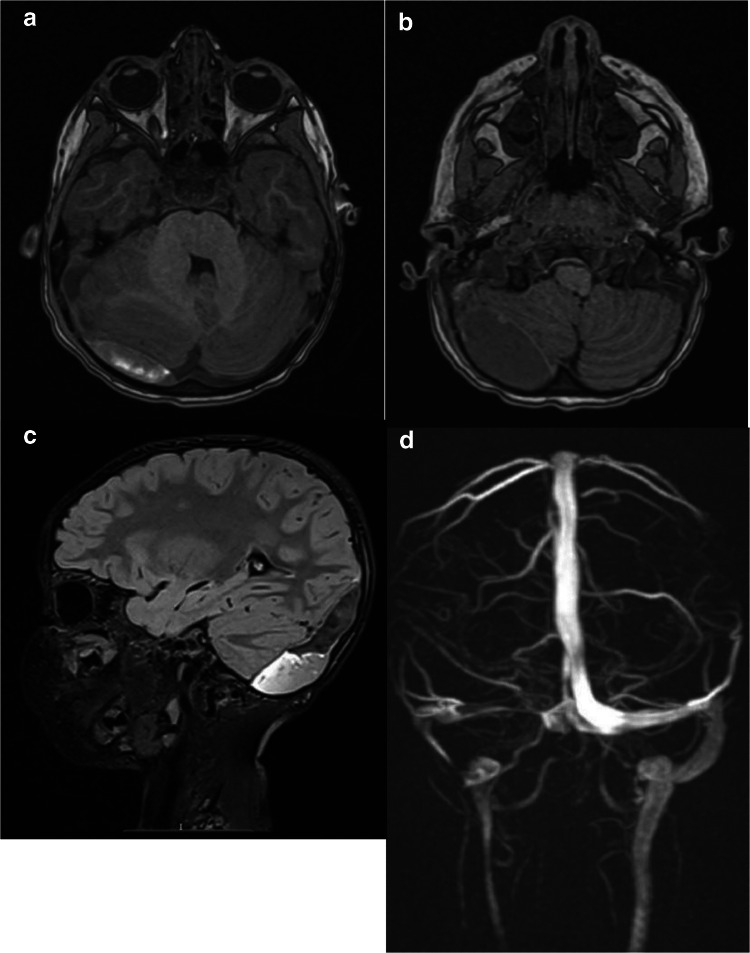
**a** and **b** T1-weighted axial and **c** T2-weighted SPACE Dark Fluid sagittal non-contrast enhanced MR images of extradural haematoma with local mass effect (4 mm midline shift to the left) and extrinsic compression of the right transverse sinus. **d** MRV representation of right sigmoid sinus thrombus resulting in near-total occlusion of this venous segment.

An MRI and MRV under GA on day 6 confirmed stable interval appearances (Fig. 3). Local mass effect within the PF was exerted with ongoing partial effacement of the fourth ventricle, pre-pontine and peri-medullary CSF spaces at the craniocervical junction suggesting an increase in ventricular calibre. Extrinsic compression and significant narrowing of the right transverse sinus were noted as it traversed the anterior convexity of the collection, though this segment of the sinus remained patent. The right sigmoid sinus thrombus resulted in near complete occlusion with a tiny pin-hole of antegrade physiological flow through the periphery of the short segment focal luminal thrombus. This remained unchanged compared to the previous CTV. The torcula and contralateral left-sided venous outflow were widely patent. Ventricular dimensions were slightly reduced compared with prior CTV on image fusion.

On day 15 of admission, a repeat CTV demonstrated a marginal increase in the size of the EDH, most notably on volumetric fusion of the imaging. Resultant increased compression of the transverse sinus and transverse sigmoid junction was identified, although the latter remained patent (maximal dimensions: 4.1 cm (AP) × 4.3 cm (TR) × 2.8 cm (CC) vs 3.8 cm × 3.9 cm × 2.2 cm previously). Increased luminal opacification confirmed improving right sigmoid sinus thrombosis. No new propagation was noted. Further interval reduction in ventricular calibre was recognised. With no new neurology and clinical stability, the risk of bleeding continued to outweigh that of thrombosis.

Soon after the diagnosis of mild haemophilia A was confirmed, the patient received 8-hourly Nuwiq (recombinant FVIII) 750 IU (50 units/kg) intravenously coupled with ferrous fumarate 100 mg twice a day for 3 days. Good haemostasis was achieved with target levels above 50% for the first 7 days. This was weaned to 500 IU TDS before further reduction to twice daily doses from exposure day (ED) (any calendar day on which a patient receives at least one dose of FVIII concentrate) four followed by once daily dosing from ED eight (10 days post injury). As per the UKHCDO guidelines, inhibitor screens (immune-mediated antibodies that inhibit infused FVIII) were undertaken on every third ED for the first 40 EDs. Treatment with Nuwiq extended over 32 EDs with a total of 43 doses administered. This was guided by radiological developments observed via serial imaging. Anticoagulation was never commenced.

Genetic testing confirmed a hemizygous pathogenic mutation of F8 (HGVS description: NM_000132.3: c.398A > G p.(Tyr133Cys) on ChrX) in association with mild haemophilia and no inhibitors reported. Further molecular analysis of extended thrombophilia genetic testing (20 genes including ADAMTS13, F2, F3, F5 and FGA) was negative. The child’s maternal grandfather was known to have haemophilia. Familial screening confirmed mild haemophilia A (baseline level: 15%) in the patient’s older brother.

## Prognosis and outcomes

The patient remained well on follow-up 2 weeks post-discharge. Repeat CTV in the outpatient setting to monitor EDH volume and assess for propagation of the thrombus confirmed an interval significant reduction in the size of the EDH (6mm depth) overlying the right cerebellum. The dural venous sinuses were noted to opacify normally. No residual or new CVT was noted confirming recanalisation and negating the need for anticoagulation therapy.

**Fig. 4:**
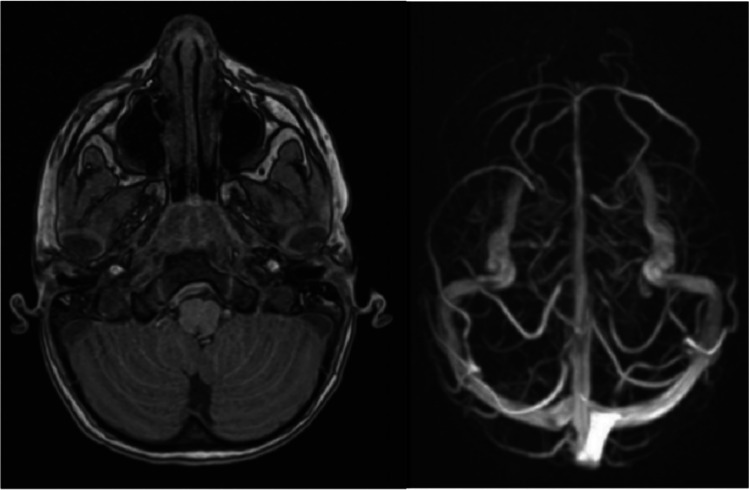
Interval MRI at 16 months confirmed complete resorption of the EDH and resolution of the CVST.

Further follow-up was arranged at 6 months in the paediatric TBI clinic. An excellent recovery was sustained with no headaches, lethargy, nausea, dizziness, altered appetite or cerebellar symptoms experienced. A repeat MRI scan was arranged to exclude chronic cerebellar insult or supratentorial injury in view of the initial mass effect and brainstem distortion secondary to the EDH. An interval MRI under GA obtained at 16 months confirmed complete resolution of the EDH with normal flow through the dural venous sinuses (Fig. 4). Follow-up arranged jointly by the neurosurgical and neuropsychological services in the TBI clinic confirmed no long-term behavioural or mood disturbances with good academic progress sustained at school.

## Discussion

**Fig. 5:**
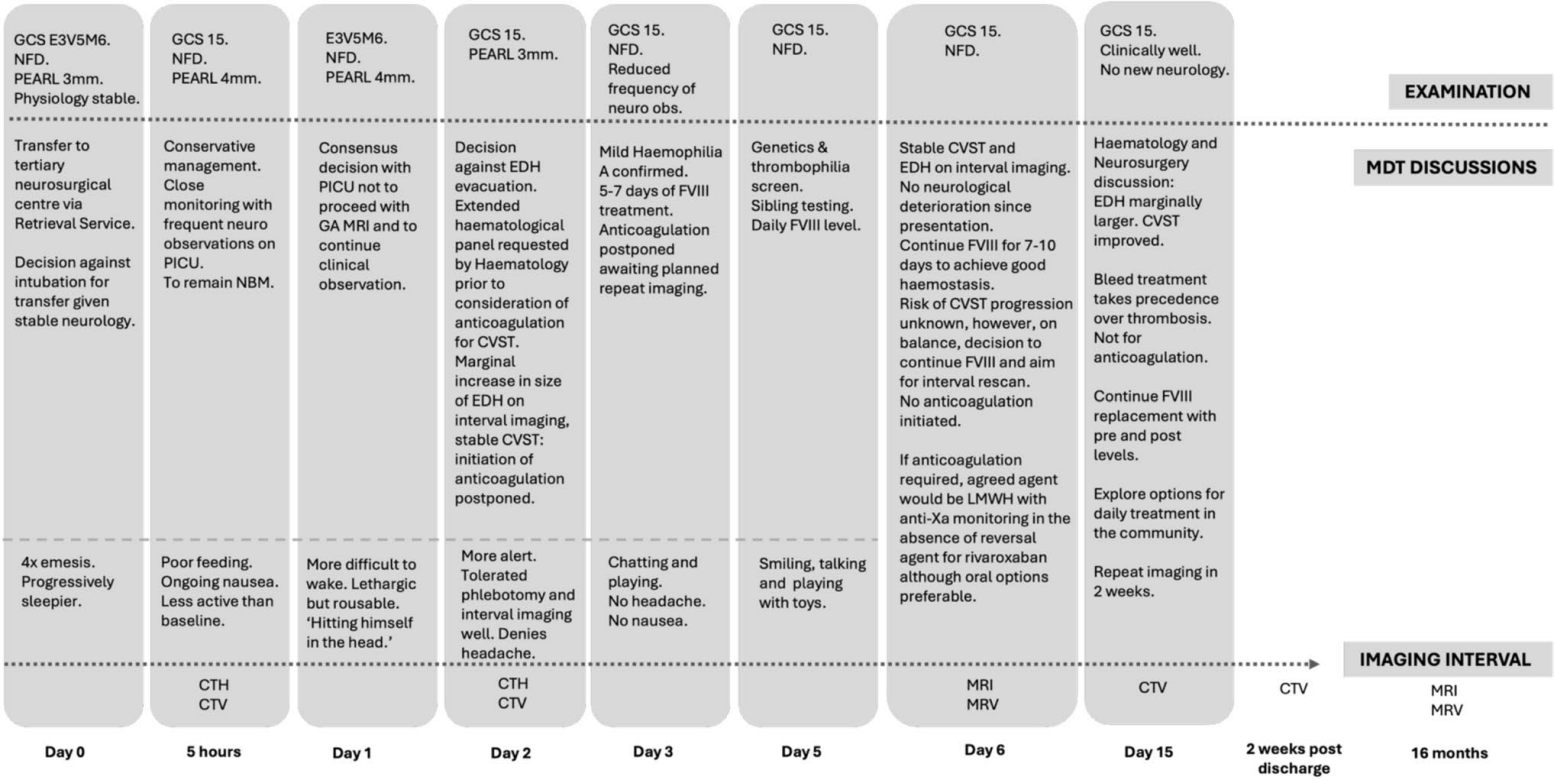
Timeline detailing the course of events from admission to outpatient follow-up. Details including evolution of symptomatology, serial examinations, MDT discussions and imaging intervals. LMWH, low molecular weight heparin; CTH, computed tomography head; CTV, computed tomography venogram; GCS, Glasgow Coma Scale; EDH, extradural haemorrhage; CVST, cerebral venous sinus thrombosis; MRI, magnetic resonance imaging; MRV, magnetic resonance venogram

To our knowledge, this is the first report of synchronous traumatic EDH and CVST with an underlying coagulopathy reported in the literature to date. There are no standardised guidelines for the management of paediatric traumatic CVST. Decisions including timing of follow-up imaging, need for anticoagulation and duration of inpatient stay for observation remain at the discretion of the local treating team (Fig. 5).

Spontaneous ICH is most often seen in paediatric cases of severe IBD less than 2 years of age with no history of prophylactic treatment [3]. There is emerging evidence of good rates of recanalisation with anticoagulation in CVST secondary to iatrogenic or traumatic aetiologies [11]. Less is assertively known in the paediatric population. However, spontaneous recanalisation with conservative management of traumatic CVST has been observed [12, 13], with suggested resolution of compression within 6 months [14–16]. Recanalisation is associated with improved clinical outcomes, although with unknown implications for clot recurrence.

If an underlying coagulopathy had not been explored in this case, the option of initiating anticoagulation to recanalise the sinus may have been instated, risking growth of the EDH. This reinforces the role of performing a clotting profile (PT, APTT and fibrinogen) in all ICHs prior to timely discussion with a paediatric haematologist if abnormalities are noted.

In this case, the CVST was thought likely secondary to damage to cerebral venous vasculature following TBI, although it would be difficult to definitively confirm an origin from the sinus or overlying occipital fracture. Contribution from the pressure effect from the EDH impacting venous return was also considered. Conservative management prioritised good haemostasis for the first 7–10 days post injury via FVIII replacement. TXA was not considered in view of the acute nature of the clot. We also did not consider merit in ICP monitoring following the transiently increased ventricular calibre in an awake patient, given the risk of bleeding.

MR imaging is a preferred modality sparing radiation for the paediatric patient. Serial scans may be required to track the clot burden, monitoring the risk of thrombus propagation. MRV can better delineate associated features including the extent of patency, hypoplastic sinus states and adjacent areas of cerebral infarct [17]. The Brain Trauma Foundation guidelines for the management of paediatric severe TBI do not advocate for routine repeat CT scans or initial follow-up without evidence of neurological deterioration or raised ICP [18]. Choice of timing for follow-up imaging is also inconsistent with reports varying between 23.5 days (median) [12] and between 3 and 12 months [19].

An argument could be made for re-establishing anticoagulation after clot evacuation. Several factors influenced the decision against neurosurgical intervention: (1) the patient remained well with no neurological compromise throughout the clinical timeline; (2) only marginal progression in the size of the EDH was demonstrated on interval imaging; (3) a significant risk of massive sinus bleeding being encountered intraoperatively persisted with a potential accompanying need to sacrifice the sinus if surgery had been pursued. Although not employed in our case nor more widely within our literature review, potential heparin reversal following initiation of anticoagulation could be associated with a paradoxical increased bleeding [20, 21], heightening the likelihood of morbidity with every acute deviation from an established coagulative state. Moreover, undertaking an emergency craniotomy over a damaged sinus in a coagulopathic patient would also pose significant intra-operative risks including air entrainment and haemorrhage less amenable to conventional techniques. On balance, a risk versus benefit decision considering these factors favoured conservative management in this case.

Whilst there is little support in the literature for the use of corticosteroids to manage cerebral oedema in TBI and obstructive hydrocephalus, there was no role for dexamethasone administration in this case [22–24].

A literature search for documented cases of traumatic EDH or SDH with or without associated CVST and haemophilia in children (aged below 18 years) yielded 13 results with 2 adult cases [25–31]. A summary of salient findings is outlined in Table 1. Of all cases associated with haemophilia, only one did not initiate FVIII replacement. Neurosurgical intervention in the form of haematoma evacuation was undertaken in 13 of 15 cases with many experiencing good post-operative recovery. Of the four cases with diagnosed CVST, none implemented anticoagulation. Three of these cases were not associated with coagulopathy but were included to demonstrate variability in practice for the management of paediatric traumatic CVST even in the absence of an IBD. Interestingly, only 1 of these 3 cases underwent surgical intervention after neurological decompensation in the adult patient.

**Table 1:** Existing reports of intracranial haematoma or CVST with associated coagulopathy with individual case context

Article information			Patient demographics		Clinical signs/symptoms	GCS/neurological status	Mechanism	Haematological diagnosis	Radiological diagnosis
Authors	Year	Number of case reports	Age	Sex					
Starobrat et al.[25]	2019	1	5 years	M	Acute thoracic and lumbar pain. No LoC, headache or seizures	Spastic paresis of lower extremities, dysesthesia, bilateral foot clonus and positive Babinski reflex	Trauma (fall from snow sled)	Haemophilia A (diagnosed at 2 months old)	EDH at T11-L2 levels
Yue & Mann[26]	1986	9	2 months	M	Dull, bulging fontanelle for 48 h	Inclusion criteria: derangement of consciousness, headache, with or without focal neurological signs	Atraumatic	Haemophilia A	Right fronto-temporal SDH
			3.5 years	M	Severe headache, dull right sensory neglect for 48 h		Trauma (fall)	Haemophilia A	Left frontal and parietal SDH
			14 years	M	Severe headache, right hemiparesis, dysphasia for 36 h		Atraumatic	Haemophilia A	Left fronto-temporal SDH
			15 years	M	Severe headache and right hemianopia for 48 h		Atraumatic	Haemophilia A	Left occipital SDH
			16 years	M	Severe headache and dull right hemiparesis for 7 days		Atraumatic	Haemophilia A	Left fronto-parietal SDH
			3 years	M	Severe headache, dull for 4 days		Atraumatic	Haemophilia A	Left hemispheric SDH
			2 years	M	Dull and vomiting for 48 h		Atraumatic	Haemophilia A	Left occipito-parietal EDH
			10 months	M	Dull and bulging fontanelle for 36 h		Atraumatic	Haemophilia A	Left frontal SDH
			14 years	M	Comatose, right hemiplegia for 24 h		Trauma (fall)	Haemophilia A	Left frontal SDH and left parietal intracerebral, communicating hydrocephalus
Beer-Furlan et al.[27]	2013	1	3 years	M	Transient LoC, conscious and irritated child on arrival	Neurological deterioration leading to intubation 40 min following arrival. Fixed dilated pupils bilaterally in theatre	Trauma (fall from height)	Haemophilia A	Right temporoparietal fracture with underlying EDH and a midline shift of 10 mm
Sahlu et al. [28]	2020	1	11 years	M	Severe headache, photophobia, double vision, emesis. Bilateral abducens nerve palsy	GCS 15. PEARL. Power MRC 5/5 upper and lower limbs bilaterally	Trauma (fall whilst playing)	Known haemophilia under regular haematology follow-up	Subacute retroclival SDH with left cerebellar and upper cervical spine extension. No clivus fracture. MRI 5 days: extra-axial collection along the anterior and postero-lateral left cerebellar hemi- sphere extending to C7 anterior upper cervical spinal canal subdural space
Cases with EDH and CVST but without coagulopathy									
Yun et al. [29]	2015	1	10 years	M	Mild intermittent headache and nausea 5 days post event	GCS 15. Normal exam	Trauma (fall from bicycle)	Normal prothrombin time, partial thromboplastin time, antithrombin III, protein C activity, and protein S activity	Linear fracture, minute epidural hematomas and pneumocephalus in the right occipital area MRI confirmed effusion and haemorrhage in right mastoid air cell. MRV confirmed right sigmoid sinus thrombus
Dobbs et al.[30]	2012	1	31 years	F	Drop in GCS, drowsiness, headache, emesis	D3 post-trauma: E3V5M6. 15 h post-op: GCS 15. 21 h post-op: GCS 3. Bilaterally dilated pupils	Trauma (fall from flight of stairs)	Nil	Right sided EDH with associated skull fracture and contre-coup contusion
Isan et al.[31]	2022	1	42 years	M	LoC; agitation and confusion	GCS 14. PEARL. Normal exam	Trauma (bicycle accident)	Nil	Right occipital EDH with non-displaced closed vertical occipital fracture extending to right transverse sinus

Article information	Haematoma volume	Haematological management	Neurosurgical management	Intra-operative blood loss	Concurrent CVST?	Outcome/post-operative status			Imaging modality
Authors						Complications within 30 days	Repeat management within 30 days	Long term deficit	
Starobrat et al.[25]	Not reported	Recombinant FVIII replacement (50U/kg) To maintain activity level 80–100 IU/dL with gradual reduction to 50 IU/dL 14 days post-op. Daily factor level assays	T11-L2 laminectomy with evacuation of haematoma and concomitant stabilisation	Not reported	No	No. No new haemorrhagic foci on immediate and 3- month post-op scans	No	Rehabilitation permitted improvement in lower limb motor function, resolution of dysesthesias and spasticity. Mobilising independently at pre-operative baseline	CT; MRI
Yue & Mann[26]	Not reported	APTT and FVIII level was assayed on admission FVIII level < 1% on admission. Group 1 patients: FVIII replacement started immediately prior to CT. Replacement targeted 100% FVIII blood level with 8-hourly infusions. An additional dose administered 1 h pre-op. Post-op day 1–3: 100% FVIII level; post-op day 4–10: 50% FVIII level. Additional dose 1 h prior to stitch removal on post-op day 7. Replacement was weaned after post-op day 10 over 2 days. APTT and FVIII levels checked twice daily pre- and post- infusion to target therapeutic level	Right fronto-temporal craniotomy	No intra-operative unusual bleeding in any of the patients	No	No	No	Good recovery at 6 months	CT
	Not reported		Left fronto-parietal craniotomy		No	No	No	Good recovery at 6 months	CT
	Not reported		Left fronto-temporal craniotomy		No	No	No	Good recovery at 6 months	CT
	Not reported		Left occipital craniotomy		No	No	No	Good recovery at 6 months	CT
	Not reported		Left fronto-parietal craniotomy		No	No	No	Good recovery at 6 months	CT
	Not reported		Left fronto-parietal craniotomy		No	No	No	Good recovery at 6 months	CT
	Not reported	APTT and FVIII level was assayed on admission FVIII level < 1% on admission. Group 2 patients: FVIII replacement commenced once haemophilia diagnosis established. Replacement targeted 100% FVIII blood level with 8-hourly infusions. An additional dose administered 1 h pre-op. Post-op day 1–3: 100% FVIII level; post-op day 4–10: 50% FVIII level	Left occipito-parietal craniotomy		No	No	No	Good recovery at 6 months	CT
			Left frontal craniotomy		No	No	No	Good recovery at 6 months	CT
	Not reported	FVIII level 5% on admission	Left frontal craniotomy and insertion of VPS		No	Peri-ventricular catheter with blockage of VPS	Revision of VPS 3 days post-op	Severely disabled at 6 months	CT
Beer-Furlan et al.[27]	Not reported		Right temporo- parietal craniotomy and EDH evacuation	Not reported	Sigmoid sinus thrombus. Subsequent propagation to right transverse and sigmoid sinuses and IJV	Worsening neurology 36 h post-op with fixed dilated right pupil. Repeat CT demonstrated venous infarction and sigmoid sinus thrombosis	Return to theatre for decompressive craniectomy with duroplasty	Haemodynamic instability with two cardiorespiratory arrests prior to subsequent death	CT
Sahlu et al. [28]	6 mm in thickness at maximum point of collection. Spinal portion spanned from foramen magnum to C7 level	Regular FVIII replacement (50U/kg for first 7 days to achieve 100% desired FVIII activity, followed by 25U/kg for next 2 weeks)	Conservative	NA	No	No	No	No. Follow-up imaging confirmed progressive, complete resolution of the hematoma. Stable clinical course whilst receiving FVIII replacement. Persistent diplopia	CT; MRI
Cases with EDH and CVST but without coagulopathy									
Yun et al. [29]	Small amounts of epidural haematomas		Conservative	NA	Right sigmoid sinus thrombus. No anticoagulation	Re-presented 10 days post presentation with vomiting. Repeat MRV confirmed spontaneous recanalisation	Conservative	Full recovery made	CT; MRI
Dobbs et al.[30]	Not reported	NA	Evacuation of EDH with insertion of ICP monitor	Not reported	Right sigmoid, transverse and straight sinus thrombus. No anticoagulation	GCS drop to 3 with seizure activity at 21 h post-op. Repeat CT demonstrated diffuse cerebral oedema and tonsillar herniation	Returned to theatre for decompressive craniectomy	Brainstem death confirmed 4 days post-trauma, treatment withdrawn 5 days post-trauma Posited that recent thrombus extension accounted for sudden deterioration	CT
Isan et al.[31]	Not reported	NA	Conservative	NA	Partial transverse sinus thrombus No anticoagulation			Further CT at 1 week, 1 month and 3 months confirmed spontaneous resorption of EDH and gradual regression of CVST	CT; MRI

*APTT*, activated partial thromboplastin time; *CNS*, central nervous system; *CSF*, cerebrospinal fluid; *CT*, computed tomography; *CVST*, cerebral venous sinus thrombosis; *D*, day; *EDH*, extradural haematoma; *FVIII*, factor VIII; *F*, female; *GCS*, Glasgow Coma Scale; *ICP*, intracranial pressure; *IJV*, internal jugular vein; *LoC*, loss of consciousness; *M*, male; *MRI*, magnetic resonance imaging; *MRV*, magnetic resonance venogram; *PEARL*, pupils equal and reactive to light; *post-op*, post-operative; *SDH*, subdural haematoma

The small number of cases identified reflects a heterogenous cohort with haematomas spanning different compartments across the neuraxis. Although most cases underwent surgical intervention, nearly all such cases were associated with preoperative neurological compromise. We also anticipate reporting bias in likely over-representation of cases with surgical management within the literature. Limited information was also available on pertinent factors including haematoma volume, blood loss and operating time. Minimal comparison could therefore be reliably drawn, warranting a renewed academic focus on the management of paediatric traumatic EDH with associated coagulopathy and CVST and a review of current guidelines.

## Conclusions

We report a rare occurrence of CVST associated with a new diagnosis of haemophilia complicating the management of traumatic PF EDH.

The role of coagulation profiles in paediatric TBI and the lack of validated guidelines for the diagnosis and management of CVST are considered. The importance of balancing anticoagulation therapy against bleeding risk is highlighted. We advocate for consideration of underlying haematological disorders in paediatric head injury disproportionate to the trauma mechanism before proceeding with empirical treatment. A role for routine performance of clotting profiles in atypical presentations of EDH despite difficult interpretation of these tests in paediatric cohorts is asserted.

Whilst the current level of evidence does not support recommendations on specific scan intervals for EDHs of a size that allows safe conservative management, we suggest frequent imaging in the first week as the risk of surgery is mitigated. Further research is warranted to support an exact scan interval. In the interim, close observation proves key to guiding a safe strategy.

## Data Availability

No datasets were generated or analysed during the current study.

## Abbreviations

CNS: Central nervous system
CT: Computed tomography
FLAIR: Fluid-attenuated inversion recovery
GCS: Glasgow Coma Scale
EDH: Extradural haematoma
CVST: Cerebral venous sinus thrombosis
APTT: Activated partial thromboplastin time
TBI: Traumatic brain injury
EDH: Extradural haematoma
SDH: Subdural haematoma
